# DNA Barcoding for the Identification of Sand Fly Species (Diptera, Psychodidae, Phlebotominae) in Colombia

**DOI:** 10.1371/journal.pone.0085496

**Published:** 2014-01-15

**Authors:** María Angélica Contreras Gutiérrez, Rafael J. Vivero, Iván D. Vélez, Charles H. Porter, Sandra Uribe

**Affiliations:** 1 Program of Study and Control of Tropical Diseases, University of Antioquia, Medellin, Colombia; 2 Division of Parasitic Diseases and Malaria, Center for Global Health, Centers for Disease Control and Prevention (CDC), Atlanta, United States of America; 3 Molecular Systematics Group, National University of Colombia, Medellin, Colombia; Natural History Museum of Denmark, Denmark

## Abstract

Sand flies include a group of insects that are of medical importance and that vary in geographic distribution, ecology, and pathogen transmission. Approximately 163 species of sand flies have been reported in Colombia. Surveillance of the presence of sand fly species and the actualization of species distribution are important for predicting risks for and monitoring the expansion of diseases which sand flies can transmit. Currently, the identification of phlebotomine sand flies is based on morphological characters. However, morphological identification requires considerable skills and taxonomic expertise. In addition, significant morphological similarity between some species, especially among females, may cause difficulties during the identification process. DNA-based approaches have become increasingly useful and promising tools for estimating sand fly diversity and for ensuring the rapid and accurate identification of species. A partial sequence of the mitochondrial cytochrome oxidase gene subunit I (COI) is currently being used to differentiate species in different animal taxa, including insects, and it is referred as a barcoding sequence. The present study explored the utility of the DNA barcode approach for the identification of phlebotomine sand flies in Colombia. We sequenced 700 bp of the COI gene from 36 species collected from different geographic localities. The COI barcode sequence divergence within a single species was <2% in most cases, whereas this divergence ranged from 9% to 26.6% among different species. These results indicated that the barcoding gene correctly discriminated among the previously morphologically identified species with an efficacy of nearly 100%. Analyses of the generated sequences indicated that the observed species groupings were consistent with the morphological identifications. In conclusion, the barcoding gene was useful for species discrimination in sand flies from Colombia.

## Introduction

The subfamily Phlebotominae Rondani, 1840, is represented in America by more than 500 species distributed among the genera *Lutzomyia* França, 1924, *Brumptomyia* França and Parrot, 1921, and *Warileya* Hertig, 1948 [1]–[3]. The *Lutzomyia* (*L.*) species are known for their role as vectors of medically important pathogens, such as the kinetoplastida protozoan of the genus *Leishmania* Ross, 1903 [4], [5], the bacterium *Bartonella bacilliformis* (Strong et al. 1907), and viruses of the families *Bunyaviridae*, *Reoviridae*, and *Rhabdoviridae* [6]–[8].

Although phlebotomine sand flies have been studied more than most other groups of insects because of their role as vectors of pathogens, the taxonomic knowledge of these insects is far from complete. Three major reviews [1], [2], [9] and the electronic documentation produced by the Walter Reed Biosystematics Unit (WRBU, http://wrbu.si.edu/) are essential tools for studying the systematics, taxonomy, and geographic distribution of this group of medically important insects in the Americas.

To date, in Colombia, 163 species of sand flies (153 *Lutzomyia*, 8 *Brumptomyia*, and 2 *Warileya* species) have been recorded. This list was obtained from taxonomic studies conducted in Colombia in different natural and endemic environments, with a primary focus on areas of leishmaniasis transmission [10]–[19].

There are approximately 13 species in the genus *Lutzomyia* that have been incriminated as vectors of *Leishmania* spp. [20] in Colombia. Since various vector species often coexist in areas of disease transmission, specific identification of the species is useful for monitoring and subsequent intervention by officials from the Vector-Borne Diseases (ETV) units [21].

Currently, the identification of species within the subfamily Phlebotominae is based on morphological recognition and morphometric analysis of a large number of structures, primarily in the head and genitalia [1], [2]. However, species identification is complicated because it requires a considerable degree of skill and taxonomic expertise. In addition, morphological identification may be limited by deterioration of samples or by improper mounting techniques.

Molecular methodology is now being used to explore taxonomic questions at different hierarchical levels [22], [23]. Ribosomal gene sequences (18SrDNA and the D2 domain in 28SrDNA) were used to reassess the relationships within the family Phlebotominae [24] and within the genus *Lutzomyia* [25]–[28]. Gene sequences with a faster rate of evolution, mitochondrial genes (e.g., cytochrome b gene and NADH Dehydrogenase 4 (ND4) gene), and nuclear genes (e.g., the complete internal transcribed spacer 2 (ITS2) and nuclear Elongation Factor 1-alpha (EF-1α) gene), have been used to resolve intraspecific and subgeneric relationships [29]–[37].

Following the proposed use of DNA sequences as a support tool for studies of biological diversity and classification [38], [39], Hebert et al. [40], [41] suggested the use and analysis of the 5′ fragment of the mitochondrial gene Cytochrome C Oxidase Subunit I (COI), called a DNA barcode. This region is of potential importance for facilitating inventories of biodiversity and performing species' identifications. The DNA barcode initiative has been well received due to the connectivity and common language of DNA sequences, allowing researchers worldwide to advance in taxonomy and systematic studies of various groups of organisms, including insect disease vectors [42]. The 5′ fragment of COI appears to be an excellent tool for studying insects of medical importance, as such study requires the quick and accurate identification of species that are present in a transmission area [28], [42], [43].

Regarding the DNA barcode, complications associated with use of this region include nuclear sequences of mitochondrial origin (NUMTs) and endosymbionts. Also, standardization of the methodology is necessary for each group studied. Furthermore, the use of a single marker for the taxonomy of a wide range of taxa is controversial [44]–[48].

Despite many demonstrations of the effectiveness of DNA barcoding in other taxa, few studies on phlebotomine sand flies in Colombia have attempted to use this methodology as a valid molecular tool for identifying species of phlebotomine sand flies [28], [49] and for revealing cryptic species [50]–[53].

In the present study, haplotypes of the barcode sequence were assigned to 148 individuals belonging to 36 phlebotomine sand fly species collected in Colombia, some of which are involved in the transmission of leishmaniasis. Specimens were obtained from sites where outbreaks of the disease have occurred. The present study evaluated the usefulness of the barcode sequence in species differentiation compared with previous morphology-based identifications.

## Materials and Methods

### Ethics Statement

Collection of sand flies was done according to the parameters of Colombian decree number 1376 http://190.147.213.68:8080/HOMEPAGE/ALEGIS_INTER/LEYES_Y_DECRETOS/2013/DECRETO_1376_de_2013.pdf which regulates the permit for collection of specimens of wild species of biological diversity for non-commercial research. No specific permits were required for this study. The sand flies were collected on private property, and permission was received from land owners prior to sampling.

### Collection of specimens

**Figure 1:**
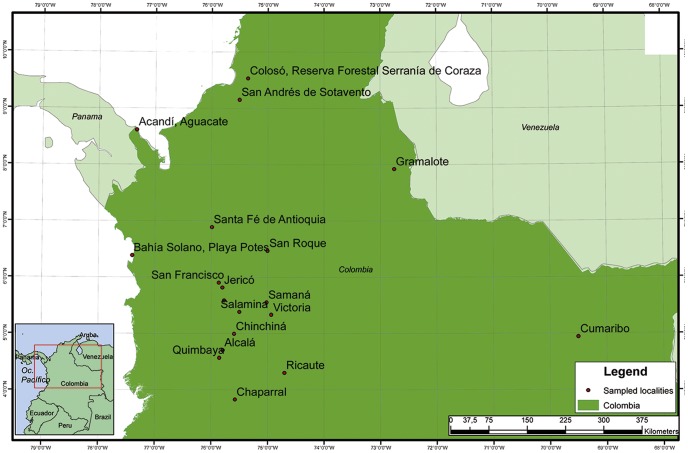
Map of the study area showing the sampling sites of sand flies collected in the present study. Site 1: Jardín; Site 2: Jerico; Site 3: San Francisco; Site 4; San Roque; Site 5: Santa Fé de Antioquia; Site 6: Chinchina; Site 7: Salamina; Site 8: Samaná; Site 9: Victoria, Site 10: Acandí; Site 11: Bahía Solano, Playa Potes; Site 12: San Andrés de Sotavento; Site 13: Ricaute; Site 14: Quimbaya; Site 15: Gramalote; Site 16: Colosó, Serranía de Coraza Forestry Reserve; Site 17: Chaparral; Site 18: Alcalá; and Site 19: Caño Ariba.

Phlebotomine sand flies were collected using CDC light traps and Shannon traps from different locations in Colombia between 2008 and 2012. Areas selected for the collections were based on previous epidemiological studies of leishmaniasis transmissionmade by the Program for the Study and Control of Tropical Diseases (PECET) (Figure 1 and table S1).The thorax and legs were removed from each specimen for DNA extraction and stored at −20°C, while the head and abdomen were processed for traditional morphological identification [1], [2], [54]. After being assigned an identification code, the remains of each specimen were preserved as a collection of voucher specimens.

### DNA extraction and polymerase chain reaction (PCR)

Total DNA (50 µL) was extracted from each sample using the Qiagen® DNeasy Blood & Tissue kit (Qiagen, Chatsworth, CA). To amplify the mitochondrial 5′ COI gene region, PCR was performed using the following thermal profile: an initial denaturation of 5 min (94°C); followed by 35 cycles at 94°C for 1 min (denaturation), 45°C for 1 min (annealing), and 72°C for 1 min (extension); and a final extension at 72°C for 10 min. Each PCR cocktail had a final reaction volume of 50 µl and contained the following: 0.3 µL of Taq DNA polymerase (5 U/µL) (Biolase®), 5 µL of 10× PCR buffer (NH_4_SO_4_), 5 µL of 2.5 mM MgCl2, 5 µL of 2 mmol/L dNTP, 2 µl of 10 µmol/L of each oligonucleotide (forward and reverse), 4 µL of the DNA template, and ddH2O to bring the volume to 50 µl. The oligonucleotides used to amplify the 700 bp COI fragment were LCO1490 and HCO2198 [41], [55].

The amplified fragments were run on an agarose gel at 1% in 1× TBE buffer (40 mM Tris-Borate, 1 mM EDTA, pH 8.0) to verify the integrity of the fragments, after which the PCR products were purified using a MultiScreen_HTS_ Vacuum Manifold (Millipore). Subsequently, the PCR products of the amplified fragments were sent for sequencing in both chain directions to Macrogen Inc. (Korea) or to the CDC (Atlanta, GA, USA).

### Data analysis

The chromatograms obtained were edited using BioEdit (http://www.mbio.ncsu.edu/bioedit/bioedit.html) [56] to generate a consensus sequence for each specimen. The DNA sequences were aligned using the Clustal W tool incorporated into MEGA v5.0 software [57]. Each sequence was compared with other insect mitochondrial genomes using BLAST (http://blast.ncbi.nlm.nih.gov/Blast.cgi) [58] to confirm the identity of the obtained fragments (the 5′ COI region). The number of haplotypes (h), polymorphic sites (s), and nucleotide diversity were determined using DnaSP v5.10 (http://www.ub.edu/dnasp) [59].

The nucleotide compositions and sequence divergences within and between species were calculated using the distance model Kimura two-parameter (K2P) [60]. A dendrogram of K2P distance Neighbor-Joining (NJ) was generated to provide a graphical representation of the clustering pattern across different species [61].

All of the sequences obtained in the present study have been submitted to GenBank (http://www.ncbi.nlm.nih.gov/genbank/). The collection locations and other information regarding the specimens, such as the GenBank accession numbers, will be available on the *BOLD Systems* (http://www.boldsystems.org) platform, as the “Phlebotominae Barcoding Initiative of the Americas - PBIN” project.

## Results

### Specimens collected

A total of 36 phlebotomine species belonging to 3 genera were collected from 11 Colombian departments (Table S1). Some of the collected species had epidemiological histories (Table 1): *Lutzomyia columbiana* (Ristorcelli and Van Ty, 1941), *Lutzomyia evansi* (Nuñez-Tovar, 1924), *Lutzomyia gomezi* (Nitzulescu, 1931), *Lutzomyia hartmanni* (Fairchild & Hertig, 1957 ), *Lutzomyia longiflocosa* Osorno, Morales Osorno and Muñoz, 1970, *Lutzomyia longipalpis* (Lutz & Neiva, 1912), *Lutzomyia nuneztovari* (Ortiz 1954), *Lutzomyia panamensis* (Shannon, 1926), *Lutzomyia pia* (Fairchild & Hertig, 1961), *Lutzomyia shannoni* (Dyar, 1929), *Lutzomyia trapidoi* (Fairchild & Hertig, 1952), *Lutzomyia youngi* Feliciangeli & Murillo, 1987, and *Lutzomyia yuilli yuilli* Young & Porter, 1972.

**Table 1:** List of sand fly species and the numbers of individuals with mitochondrial haplotypes.

Species	Number of specimens	Number of haplotypes	Number of nucleotidic divergences
*B. beaupertuyi*	1	1	0
*B. hamata*	2	2	1
*B. guimareasi*	3	2	2
*B. mesai*	2	1	0
*L. antunesi*	2	2	3
*L. barretoi majuscula*	3	2	3
*L. bifoliata*	2	1	0
*L. carpenteri*	2	1	0
*L. carrerai thula*	5	4	16
*L. cayennensis cayennensis*	1	1	0
*L. columbiana*	5	5	7
*L. coutinhoi*	3	3	6
*L. evansi*	3	3	9
*L. gomezi*	9	9	40
*L. hartmanni*	4	4	5
*L. lichyi*	1	1	0
*L. longiflocosa*	4	2	4
*L. longipalpis*	4	4	8
*L. (Lutzomyia)* sp.	2	1	0
*L. migonei*	4	2	1
*L. nuneztovari*	2	1	0
*L. panamensis*	7	6	38
*L. pia*	16	2	14
*L. reburra*	3	2	1
*L. scorzai*	9	7	28
*L. (Helcocyrtomyia)* sp.1	2	1	0
*L. shannoni*	1	1	0
*L. spinicrassa*	4	3	6
*L. sordelli*	2	1	0
*L. trapidoi*	9	9	32
*L. trinidadensis*	10	5	27
*L. triramula*	7	6	22
*L. walkeri*	2	1	0
*L. yuilli yuilli*	8	4	5
*L. youngi*	1	1	0
*W. rotundipennis*	3	2	1
**Total**	**148**	**103**	

### Sequence analysis

The 5′ fragment of the COI mitochondrial gene was amplified and sequenced for each sample (n = 148), and the total size was 700 bp. The identities of the obtained sequences corresponded with positions 16 to 716 of the COI mitochondrial gene on *Aedes aegypti* (NC_010241.1), which was used as the reference genome. This segment also corresponded with that proposed by Hebert et al. [41] as a barcode region for species identification. No insertions, deletions, or stop codons were observed indicating the absence of pseudogenes or nuclear copies of mitochondrial origin (NUMTs).The sequences from the 36 species of phlebotomine sand flies that had previously been identified based on morphology were represented by 1 to 16 individuals (Table S1). The identification numbers and GenBank accession numbers for the specimens are summarized in table S1.

During alignment at the nucleotide level, a total of 417 conserved sites and 283 variable sites were observed. The obtained COI sequences contained a large number of A+T pairs (an average of 66.3% for all codons), particularly in the third codon position (87.6%) (Table S2).

Of the 148 sequences obtained, 103 haplotypes were characterized for phlebotomine sand flies, indicating a high degree of diversity. The number of haplotypes within a species ranged from 1 to 9 (Table 1), and variable haplotypes were obtained in most species, except for *Brumptomyia mesai*, *Brumptomyia beaupertuyi*, *Lutzomyia bifoliata*, *Lutzomyia carpenteri*, *Lutzomyia nuneztovari*, *Lutzomyia sordellii*, *Lutzomyia walkeri*, and *Lutzomyia* (*Helcocyrtomyia*) sp. 1, which exhibited unique haplotypes for different specimens. The largest numbers of haplotypes were observed in *Lutzomyia gomezi* (9 haplotypes), *Lutzomyia scorzai* (7), *Lutzomyia trapidoi* (9), *Lutzomyia panamensis* (6), *Lutzomyia trinidadensis* (6), *Lutzomyia triramula* (6), *Lutzomyia columbiana* (5), and *Lutzomyia carrerai thula* (5) (Table 1).

Regarding the numbers and types of substitutions observed among different haplotypes of the same species, the results showed between 2 to 40 variable positions (Table 1), and nucleotide changes were primarily synonymous substitutions, such as transitions (purine for purine and pyrimidine for pyrimidine), at the third positions.

### Pattern of sequence divergence

The set of sequences obtained was used to evaluate efficacy of the barcoding method to delineate species. Genetic distances, as determined by the K2P intraspecies values, ranged between 0 and 6% (average intraspecific divergence = 1.6%, standard deviation = 1.38%), and maximum intraspecific divergence was observed in 8 of the 36 species (Table 2). Between species, the genetic distances showed K2P values between 9 and 26.7% (average divergence = 19%, standard deviation = 2.57%).

**Table 2:** K2P pairwise distances among Colombian sand fly species.

Species	No. of Specimens	K2P values
*B. hamata*	2	0.001
*B. guimareasi*	3	0.001–0.003
*B. mesai*	2	0.000
*L. antunesi*	2	0.004
*L. barretoi majuscula*	3	0.000
*L. bifoliata*	2	0.000
*L. carpenteri*	2	0.000
*L. carrerai thula*	5	0.001–0.018
*L. columbiana*	5	0.001–0.007
*L. coutinhoi*	3	0.001–0.009
*L. evansi*	3	0.004–0.012
*L. gomezi*	9	0.001–0.060
*L. hartmanni*	4	0.003–0.006
*L. longiflocosa*	4	0.000–0.006
*L. longipalpis*	4	0.001–0.012
*L. (Lutzomyia)* sp.	2	0.000
*L. migonei*	4	0.001–0.001
*L. nuneztovari*	2	0.000
*L. panamensis*	7	0.000–0.046
*L. pia*	16	0.000–0.021
*L. reburra*	3	0.000–0.001
*L. scorzai*	9	0.000–0.026
*L. (Helcocyrtomyia)* sp.1	2	0.000
*L. spinicrassa*	4	0.003–0.007
*L. sordelli*	2	0.001
*L. trapidoi*	9	0.000–0.030
*L. trinidadensis*	10	0.000–0.034
*L. triramula*	7	0.004–0.024
*L. walkeri*	2	0.000
*L. yuilli yuilli*	8	0.001–0.007
*W. rotundipennis*	3	0.000–0.001

**Figure 2:**
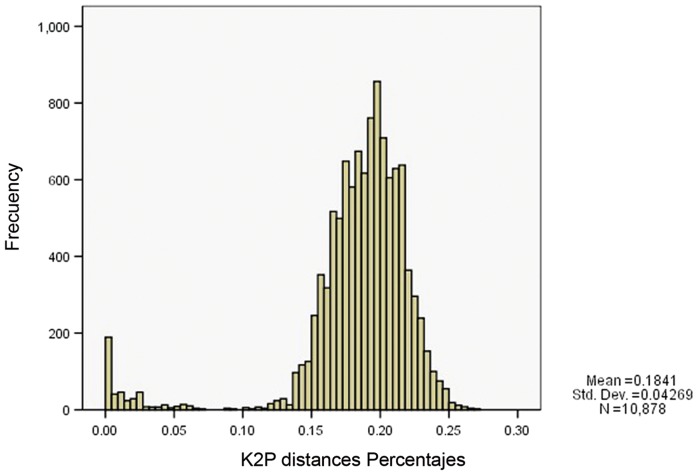
Genetic divergences (K2P distances) between COI sequences for varied taxonomic levels of Colombian sand fly species. The frequency of pairwise divergences among specimens both within species and among different species.

Given the presence of the “barcode gap” as a delimiting criterion and an indicator of species differentiation [62], [63], it was determined that the distributions of all intraspecific paired distances and the interspecific distributions of distances were not superimposed. These differences indicated genetic variations between species (Figure 2).

### Neighbor-joining dendrogram (NJ)

**Figure 3:**
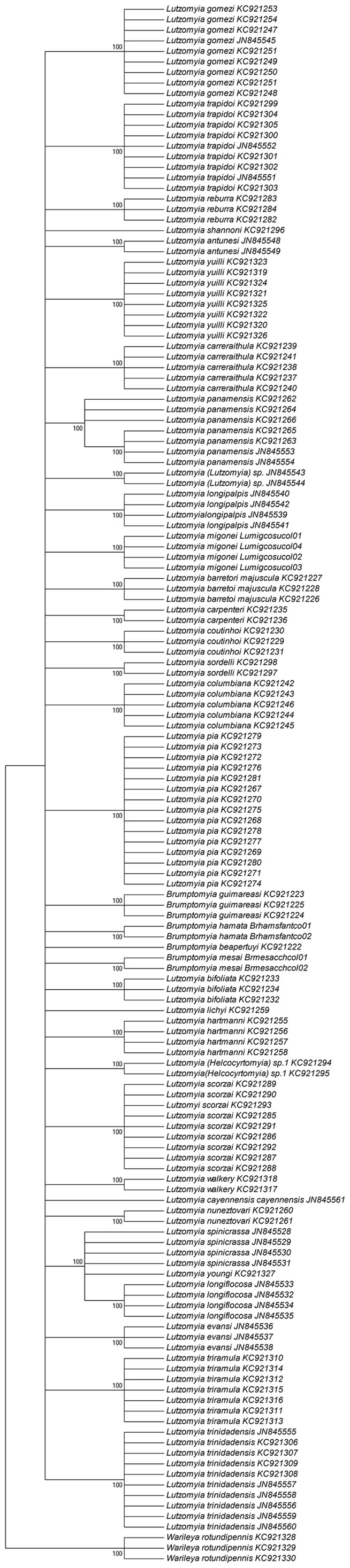
Neighbor-joining analysis of mitochondrial COI sequences of Colombian sand fly species.

NJ dendrogram (Figure 3), shows the limits representing different taxonomic groups and differentiation of species. Furthermore, specimens of the same species were always observed to be closely grouped, regardless of the collection site; for the Molecular Operational Taxonomic Units (MOTU), 36 taxonomic groups or units were identified, compared with the 36 species that were recognized by traditional taxonomy based on morphological characters. The nodes connecting the sequences of individuals from the same species were supported by high bootstrap values (99%–100%).

### Final identification

Using the divergence of K2P distances, NJ dendrogram bootstrapping, and traditional morphological approaches, we found COI sequence always corresponded with species' identifications derived from morphologic characters (Figure 3).

## Discussion

DNA barcode is a system designed to provide rapid, accurate, and automatable species-identification based on the standard analysis of short regions of genes to produce species tag [64]. The present study evaluated the efficacy of using COI barcodes for the identification of phlebotomine sand fly species in Colombia and provided a group of sequences associated with the identified species. For most species examined in the present study, a coherent matrix of DNA barcode sequences was obtained, which differentiated each species.

The 5′ region of the mitochondrial COI gene is flanked by highly conserved regions, as noted by Hebert & Gregory [64]. The absence of stop codons was consistent for all of the amplified sequences, suggesting the functionality of the COI mitochondrial sequences. These sequences were 700 bp in length, suggesting that NUMTs were not sequenced [65].

The nucleotide lengths of amplified sequences of the COI gene (700 bp) in the present study were longer than those reported in previous studies performed in Phlebotominae: 549 bp reported by Azpurua et al. [28], 681 bp by Cohnstaedt et al. [50], 689 bp by Kumar et al. [49], and 548 bp reported by Hoyos et al. [51].

Within the DNA barcode sequences in phlebotomine sand flies, high A+T contents were observed, which were within average values (68.1 to 70.7%) reported for species of the order Diptera, including suborders Nematocera and Brachycera [66], [67]. Similar values of A+T contents (66.7%) were observed in *Phlebotomus* and *Sergentomyia* in studies examining COI [28], [49]–[51]. There were no significant variations in the percentage of G+C content among the sequences of Phlebotominae from the different localities studied, and no diagnostic correlation for the percentage of G+C content was observed. The G+C content observed for the phlebotomine sand flies is similar to that of other insects and lower categories on the zoological scale [68].

In terms of codon usage, a preference for A or T termination within the triplets was clearly observed. This finding is supported by the fact that the base composition of mitochondrial DNA is highly correlated with codon usage because genes encoding mitochondrial proteins exhibit a preference for the use of codons rich in A+T. This phenomenon appears to be a characteristic of the Insecta mitochondrial genome [69], [70]. These values are similar to those observed other Diptera [66], [70]–[75] and, in particular, for species in the genus *Lutzomyia*, for which different mitochondrial gene fragments have been analyzed, including Cytochrome b (A+T = 73.09%), tRNA^serine^ (A+T = 81.2%), and ND4 (A+T = 72.5%) [31], [35], [37].

Regarding differences in the numbers and types of substitutions among different haplotypes from the same species, the results revealed 2 to 40 variable positions changes were largely synonymous substitutions of the transitional type (purine by purine and pyrimidine by pyrimidine) at third positions, which do not affect the amino acid composition of the protein. In contrast, between species, nucleotide substitution rates gradually increased in relation to genetic distances. Blouin et al. [76] suggested that nucleotide substitution patterns and rate of divergence between sites provided diagnostic information, thus, indicating the value of DNA sequences as molecular tools for differentiating species.

Aliabadian et al. [77] indicated that the success of barcode identification based on genetic distances ultimately depended on differences between intra- and interspecific divergences; ideally, there is no barcode overlap between the distributions of these two classes of distance. According to Hebert et al. [62], the barcoding gap (i.e., the difference between intra- and interspecific distances) will allow assignment of categories or species taxonomic status to specimens once barcode reference libraries are complete and available. The present study confirms that, among the taxa examined, genetic distances and barcoding gaps within a species are generally much smaller than those between individuals of different species.

The use of DNA barcoding can delineate the boundaries between species and assign taxonomic status to unknown individuals from known species. The analysis of genetic distances is one of the criteria for acquiring this information [62]. The range of intraspecific variation was between 0 and 6%, while the range of interspecific variability was between 9 and 26.7%.

Hebert et al. [41] proposed an interspecific limit of 3% for insects. The high intraspecific variability (>2%) observed between different haplotypes of *L. gomezi* (6% maximum intraspecific variability), *L. panamensis* (4.6%), and *L. trinidadensis* (3.4%) (Table 2) could suggest a population differentiation or the presence of cryptic species [51], [62]. This is a reflection of the large number of mutations observed in relation to the other sequences. Also,the wide intraspecific divergence could be related to rapid variation of the mitochondrial genome [78]; range or distribution of a species, which allows for isolation and genetic differentiation; and presence of ancestral polymorphisms, which may be conserved over time due to the aggregate population distribution characteristic of phlebotomine sand flies [79].

The low intraspecific variability observed may be the effect of sampling [80], [81]. Sample size per species ranged from 1 to 16 specimens, but only two species had more than nine individuals (Table 1). The 6% nucleotide divergence (well above the 3% level often considered an upper limit for intraspecific variation) observed for *L. gomezi* and this species' extensive geographic distribution (from Central America to South Region of Brazil) suggests the present morphospecies may be comprised of two or more cryptic species.

In the present study, we found a nucleotide divergence range between 9% and 26.7% for differentiation of the 36 species of phlebotomine sand flies identified.

In general, our results revealed that there was no overlap between the levels of intra- and interspecific divergence, the previously mentioned barcode gap, even though higher values of intraspecific divergence were observed. This separation confirms the validity and partial effectiveness of the DNA barcode sequence as a molecular identifier of species [40].

The geographical scale of sampling has a critical impact on the global application of DNA barcoding [81]. The dependency of intraspecific genetic variation on geographical scale of sampling is to be expected based on widely recognized theory and concepts such as distance decay and isolation by distance, as well as from phylogeographic studies. As a general rule, a species sampled throughout its geographical range will reveal greater genetic variation than if the variation was estimated from a single smaller region [44], [45], [63], [78], [80]–[85]. The phlebotomine sand flies species in our study were not sampled across its entire distribution range in Colombia and it will be desirable. However, when our data set was compared with published sequences in genbank, the range of intra and interspecific divergence values (0–6% and 9–26.7%) did not change. This evidence suggests accurate identification of our phlebotomine sand flies. Consequently, data derived from the COI sequence fragment were found to be helpful for species identification, and may be applied in the future for identifying species from different regions.

The NJ method is conceptually related to grouping, but does not involve a phylogenetic tool, the natural history, or the behavior of a molecular clock [86]. Combining a NJ dendrogram with a bootstrap analysis is the most appropriate method for evaluating trees using distance methods [87]. The COI NJ dendrogram confirmed that the species of a genus generally form cohesive groups.

In the present study, all MOTUs, including individuals belonging to the same species, were supported by the high bootstrap value of 100%, thus validating the consistency and usefulness of the COI barcode sequence to assign individuals to groups that corresponded to the species previously defined by specialists based on morphological features. Haplotypes from single species formed barcode groups that were clearly distinguishable from related species. As expected, some species that were represented by single individuals were not grouped with any other species (Figure 3). Including haplotypes from males and females in the analysis (Tables S1 and 1) allowed the formation of associations based on clusters when females of different species were isomorphic, for example, *L. Longiflocosa*, *L. youngi*, and *L. spinicrassa*.

The COI profile obtained for Phlebotominae confirmed the large variation that occurs in the mitochondrial genomes of these insects. Assigning species in this group required a combination of nucleotide divergence values and conventional taxonomic studies.

This study is specific to Colombia and limited to the specimens collected, it allowed for the assignment of haplotypes corresponding to species within the study area. The species included in this study represent 13 of 27 groupings proposed for Young & Duncan [1] for *Lutzomyia* genus and most of them are present in other South American countries. This is important since the increased availability of sequences for phlebotomine sand fly species will provide a more comprehensive and explicit view regarding the possibility of using the COI sequence as a molecular marker in this region.

Further study on genetic patterns and evolutionary processes will improve the ability to create a barcode-based identification system in phlebotomine sand flies and, consequently, facilitate future studies related to the biology of these insects.

## Supporting Information

Table S1 **List of sand fly species, collection sites and number of genotypes per species used in the study.**
(DOCX)

Table S2 **Sequence divergence and nucleotide composition for the sand flies genera.** The frequencies of nucleotides in sequence are presented as the total average values for all Condon positions and for each condon position separately with the accuracy to tenths of a percent.
(DOCX)
