# Supplementary material for: DNA Barcoding for the Identification of Sand Fly Species (Diptera, Psychodidae, Phlebotominae) in Colombia
Source: PLoS One. 2014 Jan 15;9(1):e85496. doi: 10.1371/journal.pone.0085496 (PMC3893204; doi:10.1371/journal.pone.0085496)
Supplement: Table S1 — List of sand fly species, collection sites and number of genotypes per species used in the study. (DOCX) [file pone.0085496.s001.docx]

Table S1. List of sand fly species, collection sites and number of genotypes per species used in the study.

| **Serial no.** | **Species** | **Sex** | **Departament** | **Collection site** | **VCRC Museum No** | **Code of sequence** |
| --- | --- | --- | --- | --- | --- | --- |
| 1 | *Brumptomyia beaupertuyi* | M | Antioquia | Site 2, Jericó | PPCO3671 | MA80_Br_beapertuyi_Jerico |
| 2 | *Brumptomyia guimaraesi* | M | Caldas | Site 6, Chinchiná | PPCO3672 | MA84_Br_guimareasi_Chin |
| 3 | *Brumptomyia guimaraesi* | F | Caldas | Site 6, Chinchiná | PPCO3673 | MA82_Br_sp_Hembra_Chin |
| 4 | *Brumptomyia guimaraesi* | F | Caldas | Site 6, Chinchiná | PPCO3674 | MA83_Br_sp_Hembra_Chin |
| 5 | *Brumptomyia hamata* | M | Antioquia | Site 3, San Francisco | PPCO3899 | Br_hamata Brhamsfantco01 |
| 6 | *Brumptomyia hamata* | M | Antioquia | Site 3, San Francisco | PPCO3900 | Br_hamata Brhamsfantco02 |
| 7 | *Brumptomyia mesai* | M | Chocó | Site 10, Acandí, Aguacate | PPCO3901 | Br_mesai Brmesacchcol01 |
| 8 | *Brumptomyia mesai* | M | Chocó | Site 10, Acandí, Aguacate | PPCO3902 | Br_mesai Brmesacchcol02 |
| 9 | *Lutzomyia antunesi* | F | Vichada | Site 19, Caño Ariba | PPCO3657 | Lu_antunesi Luantcavicol01 |
| 10 | *Lutzomyia antunesi* | F | Vichada | Site 19, Caño Ariba | PPCO3658 | Lu_antunesi Luantcavicol02 |
| 11 | *Lutzomyia barretoi majuscula* | M | Chocó | Site 11, Bahía Solano, Playa Potes | PPCO3756 | MA114_Lu_barretoi majuscula |
| 12 | *Lutzomyia barretoi majuscula* | M | Chocó | Site 11, Bahía Solano, Playa Potes | PPCO3757 | MA115_Lu_barretori majuscula |
| 13 | *Lutzomyia barretoi majuscula* | M | Chocó | Site 11, Bahía Solano, Playa Potes | PPCO3758 | MA126_Lu_barretoi majuscula |
| 14 | *Lutzomyia coutinhoi* | M | Chocó | Site 11, Bahía Solano, Playa Potes | PPCO3759 | MA116_Lu_ coutinhoi |
| 15 | *Lutzomyia coutinhoi* | M | Chocó | Site 11, Bahía Solano, Playa Potes | PPCO3760 | MA125_Lu_ coutinhoi |
| 16 | *Lutzomyia coutinhoi* | F | Chocó | Site 11, Bahía Solano, Playa Potes | PPCO3761 | MA127_Lu_ coutinhoi |
| 17 | *Lutzomyia bifoliata* | M | Caldas | Site 9, Victoria | PPCO3675 | MA46_Lu_bifoliata_Vic |
| 18 | *Lutzomyia bifoliata* | M | Caldas | Site 9, Victoria | PPCO3676 | MA47_Lu_bifoliata_Vic |
| 19 | *Lutzomyia bifoliata* | F | Caldas | Site 9, Victoria | PPCO3677 | MA48_Lu_bifoliata_Vic |
| 20 | *Lutzomyia carpenteri* | F | Caldas | Site 9, Victoria | PPCO3678 | MA60_Lu_carpenteri_Vic |
| 21 | *Lutzomyia carpenteri* | F | Caldas | Site 9, Victoria | PPCO3679 | MA61_Lu_carpenteri_Vic |
| 22 | *Lutzomyia carrerai thula* | F | Caldas | Site 6, Chinchiná | PPCO3680 | MA16_Lu_carreraithula_Chin |
| 23 | *Lutzomyia carrerai thula* | F | Caldas | Site 6, Chinchiná | PPCO3681 | MA17_Lu_carreraithula_Chin |
| 24 | *Lutzomyia carrerai thula* | F | Caldas | Site 6, Chinchiná | PPCO3682 | MA18_Lu_carreraithula_Chin |
| 25 | *Lutzomyia carrerai thula* | M | Caldas | Site 6, Chinchiná | PPCO3683 | MA19_Lu_carreraithula_Chin |
| 26 | *Lutzomyia carrerai thula* | M | Caldas | Site 6, Chinchiná | PPCO3684 | MA20_Lu_carreraithula_Chin |
| 27 | *Lutzomyia cayennensis cayennensis* | M | Antioquia | Site 5, Santa Fé de Antioquia | PPCO3670 | Lu_cayennensis Lucaysfeantcol01 |
| 28 | *Lutzomyia columbiana* | F | Caldas | Site 6, Chinchiná | PPCO3685 | MA14_Lu_columbiana_Chin |
| 29 | *Lutzomyia columbiana* | F | Caldas | Site 7, Salamina | PPCO3686 | MA15_Lu_columbiana_Chin |
| 30 | *Lutzomyia columbiana* | F | Caldas | Site 7, Salamina | PPCO3687 | MA92_Lu_columbiana_Sala |
| 31 | *Lutzomyia columbiana* | M | Caldas | Site 7, Salamina | PPCO3688 | MA93_Lu_columbiana_Sala |
| 32 | *Lutzomyia columbiana* | M |  | Site 7, Salamina | PPCO3689 | MA94_Lu_columbiana_Sala |
| 33 | *Lutzomyia evansi* | F | Córdoba | Site 12, San Andrés de Sotavento | PPCO3645 | Lu_evansi_Luevacol01 |
| 34 | *Lutzomyia evansi* | F | Córdoba | Site 12, San Andrés de Sotavento | PPCO3646 | Lu_evansi_Luevacol02 |
| 35 | *Lutzomyia evansi* | F | Córdoba | Site 12, San Andrés de Sotavento | PPCO3647 | Lu_evansi_Luevacol03 |
| 36 | *Lutzomyia gomezi* | M | Antioquia | Site 5, Santa Fé de Antioquia | PPCO3654 | Lu_gomezi_Lugomcol01 |
| 37 | *Lutzomyia gomezi* | F | Caldas | Site 9, Victoria | PPCO3690 | MA33_Lu_gomezi_Vic |
| 38 | *Lutzomyia gomezi* | M | Caldas | Site 9, Victoria | PPCO3691 | MA97_Lu_gomezi_Vic |
| 39 | *Lutzomyia gomezi* | M | Valle del Cauca | Site 18, Alcalá | PPCO3692 | MA34_Lu_gomezi_Alcala |
| 40 | *Lutzomyia gomezi* | F | Valle del Cauca | Site 18, Alcalá | PPCO3693 | MA35_Lu_gomezi_Alcala |
| 41 | *Lutzomyia gomezi* | M | Valle del Cauca | Site 18, Alcalá | PPCO3694 | MA36_Lu_gomezi_Alcala |
| 42 | *Lutzomyia gomezi* | F | Chocó | Site 11, Bahía Solano, Playa Potes | PPCO3762 | MA119_Lu_gomezi |
| 43 | *Lutzomyia gomezi* | F | Chocó | Site 11, Bahía Solano, Playa Potes | PPCO3763 | MA120_Lu_gomezi |
| 44 | *Lutzomyia gomezi* | F | Chocó | Site 11, Bahía Solano, Playa Potes | PPCO3764 | MA121_Lu_gomezi |
| 45 | *Lutzomyia hartmanni* | M | Caldas | Site 9, Victoria | PPCO3695 | MA37_Lu_hartmanni_Vic |
| 46 | *Lutzomyia hartmanni* | F | Caldas | Site 9, Victoria | PPCO3696 | MA38_Lu_hartmanni_Vic |
| 47 | *Lutzomyia hartmanni* | F | Caldas | Site 9, Victoria | PPCO3697 | MA85_Lu_hartmanni_Vic |
| 48 | *Lutzomyia hartmanni* | M | Caldas | Site 9, Victoria | PPCO3698 | MA86_Lu_hartmanni_Vic |
| 49 | *Lutzomyia lichyi* | M | Quindío | Site14, Quimbaya | PPCO3699 | MA62_Lu_lichyi_Quimbaya |
| 50 | *Lutzomyia longiflocosa* | F | Tolima | Site 17, Chaparral | PPCO3641 | Lu_longiflocosa Lulofchtocol01 |
| 51 | *Lutzomyia longiflocosa* | M | Tolima | Site 17, Chaparral | PPCO3642 | Lu_longiflocosa Lulofchtocol02 |
| 52 | *Lutzomyia longiflocosa* | M | Tolima | Site 17, Chaparral | PPCO3643 | Lu_longiflocosa Lulofchtocol03 |
| 53 | *Lutzomyia longiflocosa* | F | Tolima | Site 17, Chaparral | PPCO3644 | Lu_longiflocosa Lulofchtocol04 |
| 54 | *Lutzomyia longipalpis* | M | Cundinamarca | Site 13, Ricaute | PPCO3648 | Lu_longipalpis Lulonricucol01 |
| 55 | *Lutzomyia longipalpis* | F | Cundinamarca | Site 13, Ricaute | PPCO3649 | Lu_longipalpis Lulonricucol02 |
| 56 | *Lutzomyia longipalpis* | M | Cundinamarca | Site 13, Ricaute | PPCO3650 | Lu_longipalpis Lulonricucol03 |
| 57 | *Lutzomyia longipalpis* | M | Cundinamarca | Site 13, Ricaute | PPCO3651 | Lu_longipalpis Lulonricucol04 |
| 58 | *Lutzomyia* (*Lutzomyia*) sp. | M | Antioquia | Site 4, San Roque | PPCO3652 | Lu_(Lutzomyia)_sp Lubifcol01 |
| 59 | *Lutzomyia* (*Lutzomyia*) sp. | M | Antioquia | Site 4, San Roque | PPCO3653 | Lu_(Lutzomyia)_sp Lubifcol02 |
| 60 | *Lutzomyia migonei* | M | Sucre | Site 16, Colosó, Reserva Forestal Serranía de Coraza | PPCO3903 | Lu_migonei Lumigcosucol01 |
| 61 | *Lutzomyia migonei* | M | Sucre | Site 16, Colosó, Reserva Forestal Serranía de Coraza | PPCO3904 | Lu_migonei Lumigcosucol02 |
| 62 | *Lutzomyia migonei* | M | Sucre | Site 16, Colosó, Reserva Forestal Serranía de Coraza | PPCO3905 | Lu_migonei Lumigcosucol03 |
| 63 | *Lutzomyia migonei* | M | Sucre | Site 16, Colosó, Reserva Forestal Serranía de Coraza | PPCO3906 | Lu_migonei Lumigcosucol04 |
| 64 | *Lutzomyia nuneztovari* | F | Caldas | Site 7, Salamina | PPCO3700 | MA42_Lu_nuneztovari_Sala |
| 65 | *Lutzomyia nuneztovari* | M | Caldas | Site 7, Salamina | PPCO3701 | MA50_Lu_nuneztovari_Sala |
| 66 | *Lutzomyia panamensis* | F | Antioquia | Site 3, San Francisco | PPCO3662 | Lu_panamensis Lupansfantcol01 |
| 67 | *Lutzomyia panamensis* | F | Antioquia | Site 3, San Francisco | PPCO3663 | Lu_panamensis Lupansfantcol02 |
| 68 | *Lutzomyia panamensis* | F | Caldas | Site 9, Victoria | PPCO3702 | MA66_Lu_panamensis_Vic |
| 69 | *Lutzomyia panamensis* | F | Caldas | Site 9, Victoria | PPCO3703 | MA67_Lu_panamensis_Vic |
| 70 | *Lutzomyia panamensis* | F | Caldas | Site 9, Victoria | PPCO3704 | MA68_Lu_panamensis_Vic |
| 71 | *Lutzomyia panamensis* | M | Caldas | Site 9, Victoria | PPCO3705 | MA69_Lu_panamensis_Vic |
| 72 | *Lutzomyia panamensis* | M | Caldas | Site 9, Victoria | PPCO3706 | MA89_Lu_panamensis_Vic |
| 73 | *Lutzomyia pia* | F | Caldas | Site 6, Chinchiná | PPCO3707 | AM1_Lu_pia_Chin |
| 74 | *Lutzomyia pia* | F | Caldas | Site 6, Chinchiná | PPCO3708 | AM2_Lu_pia_Chin |
| 75 | *Lutzomyia pia* | F | Caldas | Site 6, Chinchiná | PPCO3709 | AM3_Lu_pia_Chin |
| 76 | *Lutzomyia pia* | F | Caldas | Site 6, Chinchiná | PPCO3710 | AM4_Lu_pia_Chin |
| 77 | *Lutzomyia pia* | M | Caldas | Site 6, Chinchiná | PPCO3711 | MA5_Lu_pia_Chin |
| 78 | *Lutzomyia pia* | F | Caldas | Site 6, Chinchiná | PPCO3712 | AM6_Lu_pia_Chin |
| 79 | *Lutzomyia pia* | F | Caldas | Site 6, Chinchiná | PPCO3713 | MA7_Lu_pia_Chin |
| 80 | *Lutzomyia pia* | F | Caldas | Site 6, Chinchiná | PPCO3714 | MA9_Lu_pia_Chin |
| 81 | *Lutzomyia pia* | F | Caldas | Site 7, Salamina | PPCO3715 | MA10_Lu_pia_Sala |
| 82 | *Lutzomyia pia* | F | Caldas | Site 7, Salamina | PPCO3716 | MA11_Lu_pia_Sala |
| 83 | *Lutzomyia pia* | M | Caldas | Site 7, Salamina | PPCO3717 | MA12_Lu_pia_Sala |
| 84 | *Lutzomyia pia* | M | Caldas | Site 7, Salamina | PPCO3718 | MA13_Lu_pia_Sala |
| 85 | *Lutzomyia pia* | M | Caldas | Site 7, Salamina | PPCO3719 | MA53_Lu_pia_Sala |
| 86 | *Lutzomyia pia* | M | Caldas | Site 7, Salamina | PPCO3720 | MA54_Lu_pia_Sala |
| 87 | *Lutzomyia pia* | F | Caldas | Site 7, Salamina | PPCO3721 | MA98_Lu_pia_Sala |
| 88 | *Lutzomyia reburra* | F | Chocó | Site 11, Bahía Solano, Playa Potes | PPCO3765 | MA117_Lu_reburra |
| 89 | *Lutzomyia reburra* | F | Chocó | Site 11, Bahía Solano, Playa Potes | PPCO3766 | MA118_Lu_reburra |
| 90 | *Lutzomyia reburra* | M | Chocó | Site 11, Bahía Solano, Playa Potes | PPCO3767 | MA124_Lu_reburra |
| 91 | *Lutzomyia scorzai* | M | Antioquia | Site 1, El Jardín | PPCO3722 | MA70_Lu_scorzai_Jardin |
| 92 | *Lutzomyia scorzai* | M | Antioquia | Site 1, El Jardín | PPCO3723 | MA71_Lu_scorzai_Jardin |
| 93 | *Lutzomyia scorzai* | M | Antioquia | Site 1, El Jardín | PPCO3724 | MA72_Lu_scorzai_Jardin |
| 94 | *Lutzomyia scorzai* | M | Antioquia | Site 1, El Jardín | PPCO3725 | MA79_Lu_scorzai_Jardin |
| 95 | *Lutzomyia scorzai* | M | Antioquia | Site 1, El Jardín | PPCO3726 | MA96_Lu_scorzai_Jardin |
| 96 | *Lutzomyia scorzai* | F | Antioquia | Site 1, El Jardín | PPCO3758 | MA73_Lu_(Helcocyrtomyia)_sp.2_Jardin |
| 97 | *Lutzomyia scorzai* | F | Antioquia | Site 1, El Jardín | PPCO3759 | MA74_Lu_(Helcocyrtomyia)_sp.2_Jardin |
| 98 | *Lutzomyia scorzai* | F | Antioquia | Site 1, El Jardín | PPCO3760 | MA75_Lu_(Helcocyrtomyia)_sp.2_Jardin |
| 99 | *Lutzomyia scorzai* | F | Antioquia | Site 1, El Jardín | PPCO3761 | MA76_Lu_(Helcocyrtomyia)_sp.2_Jardin |
| 100 | *Lutzomyia shannoni* | M | Quindío | Site 14, Quimbaya | PPCO3729 | MA63_Lu_shannoni_Quimbaya |
| 101 | *Lutzomyia spinicrassa* | M | Santander | Site 15, Gramalote | PPCO3637 | Lu_spinicrassa Luspcol01 |
| 102 | *Lutzomyia spinicrassa* | M | Santander | Site 15, Gramalote | PPCO3638 | Lu_spinicrassa Luspcol02 |
| 103 | *Lutzomyia spinicrassa* | M | Santander | Site 15, Gramalote | PPCO3639 | Lu_spinicrassa Luspcol03 |
| 104 | *Lutzomyia spinicrassa* | M | Santander | Site 15, Gramalote | PPCO3640 | Lu_spinicrassa Luspcol04 |
| 105 | *Lutzomyia sordelli* | F | Caldas | Site 9, Victoria | PPCO3730 | MA64_Lu_sordelli_Vic |
| 106 | *Lutzomyia sordelli* | F | Caldas | Site 9, Victoria | PPCO3731 | MA65_Lu_sordelli_Vic |
| 107 | *Lutzomyia trapidoi* | F | Antioquia | Site 3, San Francisco | PPCO3660 | Lu_trapidoi Lutrasfantcol01 |
| 108 | *Lutzomyia trapidoi* | F | Antioquia | Site 3, San Francisco | PPCO3661 | Lu_trapidoi Lutrasfantcol02 |
| 109 | *Lutzomyia trapidoi* | M | Caldas | Site 9, Victoria | PPCO3732 | MA22_Lu_trapidoi_Vic |
| 110 | *Lutzomyia trapidoi* | M | Caldas | Site 9, Victoria | PPCO3733 | MA23_Lu_trapidoi_Vic |
| 111 | *Lutzomyia trapidoi* | M | Caldas | Site 9, Victoria | PPCO3734 | MA24_Lu_trapidoi_Vic |
| 112 | *Lutzomyia trapidoi* | F | Caldas | Site 9, Victoria | PPCO3735 | MA25_Lu_trapidoi_Vic |
| 113 | *Lutzomyia trapidoi* | F | Caldas | Site 9, Victoria | PPCO3768 | MA26_Lu_trapidoi_Vic |
| 114 | *Lutzomyia trapidoi* | M | Caldas | Site 8, Samaná | PPCO3769 | MA32_Lu_trapidoi_Vic |
| 115 | *Lutzomyia trapidoi* | F | Chocó | Site 11, Bahía Solano, Playa Potes | PPCO3770 | MA123_Lu_trapidoi |
| 116 | *Lutzomyia trinidadensis* | F |  |  | PPCO3664 | Lu_trinidadensis Lutricol01 |
| 117 | *Lutzomyia trinidadensis* | F | Antioquia | Site 3, San Francisco | PPCO3665 | Lu_trinidadensis Lutrisfantcol01 |
| 118 | *Lutzomyia trinidadensis* | F | Antioquia | Site 3, San Francisco | PPCO3666 | Lu_trinidadensis Lutrisfantcol02 |
| 119 | *Lutzomyia trinidadensis* | F | Antioquia | Site 3, San Francisco | PPCO3667 | Lu_trinidadensis Lutrisfantcol03 |
| 120 | *Lutzomyia trinidadensis* | M | Antioquia | Site 3, San Francisco | PPCO3668 | Lu_trinidadensis Lutrisfantcol04 |
| 121 | *Lutzomyia trinidadensis* | M | Antioquia | Site 3, San Francisco | PPCO3669 | Lu_trinidadensis Lutrisfantcol05 |
| 122 | *Lutzomyia trinidadensis* | M | Chocó | Site 11, Bahía Solano, Playa Potes | PPCO3771 | MA109_Lu_trinidadensis |
| 123 | *Lutzomyia trinidadensis* | M | Chocó | Site 11, Bahía Solano, Playa Potes | PPCO3772 | MA110_Lu_trinidadensis |
| 124 | *Lutzomyia trinidadensis* | H | Chocó | Site 11, Bahía Solano, Playa Potes | PPCO3736 | MA111_Lu_trinidadensis |
| 125 | *Lutzomyia trinidadensis* | H | Chocó | Site 11, Bahía Solano, Playa Potes | PPCO3737 | MA112_Lu_trinidadensis |
| 126 | *Lutzomyia triramula* | M | Caldas | Site 9, Victoria | PPCO3738 | MA21_Lu_triramula_Vic |
| 127 | *Lutzomyia triramula* | M | Caldas | Site 9, Victoria | PPCO3739 | MA27_Lu_triramula_Vic |
| 128 | *Lutzomyia triramula* | M | Caldas | Site 9, Victoria | PPCO3740 | MA28_Lu_triramula_Vic |
| 129 | *Lutzomyia triramula* | F | Caldas | Site 9, Victoria | PPCO3741 | MA29_Lu_triramula_Vic |
| 130 | *Lutzomyia triramula* | F | Caldas | Site 9, Victoria | PPCO3773 | MA30_Lu_triramula_Vic |
| 131 | *Lutzomyia triramula* | F | Caldas | Site 9, Victoria | PPCO3742 | MA88_Lu_triramula_Vic |
| 132 | *Lutzomyia triramula* | M | Chocó | Site 11, Bahía Solano, Playa Potes | PPCO3743 | MA122_Lu_triramula |
| 133 | *Lutzomyia walkeri* | F | Caldas | Site 9, Victoria | PPCO3745 | MA81_Lu_walkery_Vic |
| 134 | *Lutzomyia walkeri* | M | Caldas | Site 8, Samaná | PPCO3746 | MA58_Lu_walkery_Vic |
| 135 | *Lutzomyia yuilli* | F | Caldas | Site 9, Victoria | PPCO3747 | MA39_Lu_yuilli_Vic |
| 136 | *Lutzomyia yuilli* | F | Caldas | Site 9, Victoria | PPCO3748 | MA40_Lu_yuilli_Vic |
| 137 | *Lutzomyia yuilli* | F | Caldas | Site 9, Victoria | PPCO3749 | MA41_Lu_yuilli_Vic |
| 138 | *Lutzomyia yuilli* | M | Caldas | Site 9, Victoria | PPCO3750 | MA43_Lu_yuilli_Vic |
| 139 | *Lutzomyia yuilli* | M | Caldas | Site 9, Victoria | PPCO3751 | MA44_Lu_yuilli_Vic |
| 140 | *Lutzomyia yuilli* | F | Caldas | Site 8, Samaná | PPCO3752 | MA45_Lu_yuilli_Vic |
| 141 | *Lutzomyia yuilli* | F | Caldas | Site 8, Samaná | PPCO3744 | MA51_Lu_yuilli_Vic |
| 142 | *Lutzomyia yuilli* | M | Caldas | Site 8, Samaná | PPCO3756 | MA52_Lu_yuilli_Vic |
| 143 | *Lutzomyia youngi* | M | Caldas | Site 7, Salamina | PPCO3757 | MA91_Lu_youngi_Sala |
| 144 | *Lutzomyia (Helcocyrtomyia)* sp. 1 | M | Antioquia | Site 1, El Jardín | PPCO3727 | MA77_Lu_(Helcocyrtomyia)_sp.1_Jardin |
| 145 | *Lutzomyia (Helcocyrtomyia)* sp. 1 | M | Antioquia | Site 1, El Jardín | PPCO3728 | MA78_Lu_(Helcocyrtomyia)_sp.1_Jardin |
| 146 | *Warileya rotundipennis* | F | Antioquia | Site 1, El Jardín | PPCO3753 | MA56_Wa_rotundipennis_Jeri |
| 147 | *Warileya rotundipennis* | M | Antioquia | Site 1, El Jardín | PPCO3754 | MA57_Wa_rotundipennis_Jeri |
| 148 | *Warileya rotundipennis* | F | Antioquia | Site 1, El Jardín | PPCO3755 | MA95_Wa_rotundipennis_Jeri |
