# Supplementary material for: DNA Barcoding for the Identification of Sand Fly Species (Diptera, Psychodidae, Phlebotominae) in Colombia
Source: PLoS One. 2014 Jan 15;9(1):e85496. doi: 10.1371/journal.pone.0085496 (PMC3893204; doi:10.1371/journal.pone.0085496)
Supplement: Table S2 — Sequence divergence and nucleotide composition for the sand flies genera. The frequencies of nucleotides in sequence are presented as the total average values for all Condon positions and for each condon position separately with the accuracy to tenths of a percent. (DOCX) [file pone.0085496.s002.docx]

Table S2. Sequence divergence and nucleotide composition for the sand flies genera. The frequencies of nucleotides in sequence are presented as the total average values for all Condon positions and for each condon position separately with the accuracy to tenths of a percent.

| **Especie** | **Total** | | | | | **First codon position** | | | | | **Second codon position** | | | | | **Third codon position** | | | | |
| --- | --- | --- | --- | --- | --- | --- | --- | --- | --- | --- | --- | --- | --- | --- | --- | --- | --- | --- | --- | --- |
|  | **T%** | **C%** | **A%** | **G%** | **A+T%** | **T%** | **C%** | **A%** | **G%** | **A+T%** | **T%** | **C%** | **A%** | **G%** | **A+T%** | **T%** | **C%** | **A%** | **G%** | **A+T%** |
| *B. beapertuyi* | 34.9 | 19.1 | 29.3 | 16.7 | 64.2 | 23.2 | 18.9 | 27.5 | 30.5 | 50.7 | 41.6 | 25.8 | 15.5 | 17.2 | 57.1 | 39.7 | 12.8 | 44.9 | 2.6 | 84.6 |
| *B. hamata* | 36.0 | 18.4 | 28.8 | 16.9 | 64.8 | 24.0 | 18.0 | 27.5 | 30.5 | 51.5 | 41.6 | 25.8 | 15.5 | 17.2 | 57.1 | 42.3 | 11.3 | 43.4 | 3.0 | 85.7 |
| *B. guimareasi* | 37.4 | 17.7 | 28.4 | 16.5 | 65.8 | 23.6 | 18.5 | 27.5 | 30.5 | 51.1 | 41.6 | 25.8 | 15.5 | 17.2 | 57.1 | 47.0 | 8.8 | 42.2 | 2.0 | 89.2 |
| *B. mesai* | 35.2 | 19.1 | 30.2 | 15.5 | 65.4 | 21.9 | 18.6 | 29.5 | 30.1 | 51.4 | 43.2 | 27.9 | 13.7 | 15.3 | 56.9 | 40.4 | 10.9 | 47.5 | 1.1 | 87.9 |
| *L. antunesi* | 38.0 | 16.2 | 30.0 | 15.9 | 68.0 | 27.3 | 15.7 | 27.9 | 29.2 | 55.2 | 42.1 | 25.3 | 15.5 | 17.2 | 57.6 | 44.7 | 7.7 | 46.4 | 1.3 | 91.1 |
| *L. barretoi majuscula* | 36.2 | 18.3 | 30.2 | 15.2 | 66.4 | 24.9 | 18.0 | 28.8 | 28.3 | 53.7 | 42.5 | 24.9 | 15.5 | 17.2 | 58.0 | 41.3 | 12.1 | 46.3 | 0.3 | 87.6 |
| *L. coutinhoi* | 38.1 | 16.7 | 28.2 | 17.0 | 66.3 | 25.8 | 17.2 | 27.9 | 29.2 | 53.7 | 42.1 | 25.3 | 15.5 | 17.2 | 57.6 | 46.5 | 7.6 | 41.3 | 4.7 | 87.8 |
| *L. bifoliata* | 39.1 | 17.1 | 27.6 | 16.1 | 66.7 | 23.2 | 19.3 | 28.3 | 29.2 | 51.5 | 42.1 | 25.3 | 15.5 | 17.2 | 57.6 | 52.1 | 6.8 | 38.9 | 2.1 | 91.0 |
| *L. carpenteri* | 36.6 | 18.7 | 28.4 | 16.3 | 65.0 | 25.3 | 17.6 | 28.3 | 28.8 | 53.6 | 42.1 | 25.3 | 15.5 | 17.2 | 57.6 | 42.3 | 13.2 | 41.5 | 3.0 | 83.8 |
| *L. carreraithula* | 39.1 | 15.7 | 28.8 | 16.4 | 67.9 | 27.5 | 14.6 | 28.3 | 29.6 | 55.8 | 42.1 | 25.8 | 15.0 | 17.2 | 57.1 | 48.0 | 6.7 | 42.9 | 2.4 | 90.9 |
| *L. cayennensis cayennensis* | 38.0 | 17.0 | 28.1 | 16.9 | 66.1 | 25.3 | 17.2 | 28.3 | 29.2 | 53.6 | 42.5 | 24.9 | 15.5 | 17.2 | 58.0 | 46.2 | 9.0 | 40.6 | 4.3 | 86.8 |
| *L. columbiana* | 35.3 | 19.4 | 28.9 | 16.4 | 64.2 | 24.8 | 17.7 | 28.3 | 29.2 | 53.1 | 42.1 | 25.3 | 15.5 | 17.2 | 57.6 | 39.1 | 15.2 | 42.8 | 2.9 | 81.9 |
| *L. evansi* | 39.7 | 16.9 | 26.9 | 16.6 | 66.6 | 26.2 | 16.3 | 27.9 | 29.6 | 54.1 | 42.1 | 25.3 | 15.5 | 17.2 | 57.6 | 50.7 | 9.1 | 37.2 | 3.0 | 87.9 |
| *L. gomezi* | 37.0 | 18.9 | 27.9 | 16.2 | 64.9 | 24.1 | 18.4 | 27.4 | 30.1 | 51.5 | 42.1 | 25.3 | 15.5 | 17.2 | 57.6 | 44.9 | 13.0 | 40.8 | 1.3 | 85.7 |
| *L. hartmanni* | 38.9 | 16.8 | 28.4 | 16.0 | 67.2 | 24.9 | 17.6 | 28.0 | 29.5 | 52.9 | 42.1 | 25.3 | 15.5 | 17.2 | 57.6 | 49.6 | 7.4 | 41.6 | 1.5 | 91.2 |
| *L. lichyi* | 38.0 | 18.3 | 28.0 | 15.7 | 66.0 | 24.0 | 18.5 | 29.6 | 27.9 | 53.6 | 42.1 | 25.3 | 15.5 | 17.2 | 57.6 | 47.9 | 11.1 | 38.9 | 2.1 | 86.8 |
| *L. longiflocosa* | 39.3 | 16.4 | 28.1 | 16.2 | 67.4 | 28.3 | 14.2 | 27.9 | 29.6 | 56.2 | 42.1 | 25.3 | 15.5 | 17.2 | 57.6 | 47.5 | 9.7 | 40.9 | 1.8 | 88.5 |
| *L. longipalpis* | 38.5 | 17.2 | 27.2 | 17.0 | 65.7 | 27.0 | 15.6 | 27.9 | 29.5 | 54.9 | 42.1 | 25.3 | 15.5 | 17.2 | 57.6 | 46.5 | 10.8 | 38.3 | 4.5 | 84.8 |
| *L. (Lutzomyia)* sp. | 38.7 | 16.0 | 29.6 | 15.7 | 68.3 | 27.5 | 15.9 | 27.0 | 29.6 | 54.5 | 42.1 | 26.2 | 15.5 | 16.3 | 57.6 | 46.6 | 6.0 | 46.2 | 1.3 | 92.8 |
| *L. migonei* | 37.9 | 15.0 | 31.1 | 16.1 | 69.0 | 28.3 | 14.2 | 27.9 | 29.6 | 56.2 | 42.1 | 25.3 | 15.5 | 17.2 | 57.6 | 43.2 | 5.6 | 49.8 | 1.5 | 93.0 |
| *L. nuneztovari* | 36.1 | 19.0 | 29.0 | 15.9 | 65.1 | 24.5 | 17.6 | 28.8 | 29.2 | 53.3 | 42.1 | 25.3 | 15.5 | 17.2 | 57.6 | 41.9 | 14.1 | 42.7 | 1.3 | 84.6 |
| *L. panamensis* | 40.0 | 14.9 | 28.8 | 16.3 | 68.8 | 26.7 | 16.2 | 27.7 | 29.4 | 54.4 | 42.2 | 25.2 | 15.5 | 17.2 | 57.7 | 51.2 | 3.2 | 43.2 | 2.4 | 94.4 |
| *L. pia* | 38.9 | 16.3 | 29.3 | 15.6 | 68.2 | 25.3 | 16.8 | 29.6 | 28.3 | 54.9 | 42.1 | 25.3 | 15.5 | 17.2 | 57.6 | 49.0 | 6.9 | 42.7 | 1.3 | 91.7 |
| *L. reburra* | 38.4 | 16.3 | 29.3 | 16.0 | 67.7 | 27.0 | 15.9 | 27.6 | 29.5 | 54.6 | 42.1 | 25.3 | 15.5 | 17.2 | 57.6 | 46.2 | 7.7 | 44.9 | 1.3 | 91.1 |
| *L. scorzai* | 37.0 | 19.5 | 26.4 | 17.1 | 63.4 | 22.9 | 19.5 | 27.8 | 29.7 | 50.7 | 42.1 | 25.3 | 15.5 | 17.2 | 57.6 | 46.0 | 13.7 | 36.0 | 4.4 | 81.9 |
| *L. (Helcocyrtomyia)* sp.1 | 37.6 | 18.4 | 26.9 | 17.1 | 64.5 | 25.3 | 17.2 | 27.9 | 29.6 | 53.2 | 42.1 | 25.3 | 15.5 | 17.2 | 57.6 | 45.3 | 12.8 | 37.2 | 4.7 | 82.5 |
| *L. shannoni* | 36.7 | 19.4 | 27.6 | 16.3 | 64.3 | 24.5 | 18.5 | 27.5 | 29.6 | 52.0 | 42.1 | 25.3 | 15.5 | 17.2 | 57.6 | 43.6 | 14.5 | 39.7 | 2.1 | 83.3 |
| *L. spinicrassa* | 38.6 | 16.4 | 27.6 | 17.5 | 66.2 | 27.4 | 15.0 | 28.0 | 29.6 | 55.4 | 42.1 | 25.3 | 15.5 | 17.2 | 57.6 | 46.4 | 8.8 | 39.2 | 5.7 | 85.6 |
| *L. sordelli* | 36.8 | 17.9 | 29.1 | 16.3 | 65.9 | 24.3 | 18.5 | 27.7 | 29.6 | 52.0 | 42.1 | 25.3 | 15.5 | 17.2 | 57.6 | 44.0 | 9.8 | 44.0 | 2.1 | 88.0 |
| *L. trapidoi* | 36.8 | 18.8 | 27.7 | 16.8 | 64.5 | 25.9 | 17.1 | 27.5 | 29.6 | 53.4 | 42.0 | 25.3 | 15.6 | 17.1 | 57.6 | 42.5 | 13.9 | 39.9 | 3.7 | 82.5 |
| *L. trinidadensis* | 36.0 | 19.0 | 28.7 | 16.5 | 64.6 | 22.3 | 19.3 | 28.3 | 30.0 | 50.6 | 42.1 | 25.8 | 15.0 | 17.2 | 57.1 | 43.5 | 11.8 | 42.5 | 2.2 | 86.0 |
| *L. triramula* | 37.2 | 17.7 | 28.7 | 16.4 | 65.9 | 26.9 | 15.6 | 27.9 | 29.6 | 54.8 | 42.1 | 25.3 | 15.5 | 17.2 | 57.6 | 42.5 | 12.2 | 42.6 | 2.6 | 85.1 |
| *L. walkery* | 36.6 | 18.1 | 29.0 | 16.3 | 65.6 | 26.2 | 16.3 | 27.9 | 29.6 | 54.1 | 42.1 | 25.3 | 15.5 | 17.2 | 57.6 | 41.5 | 12.8 | 43.6 | 2.1 | 85.1 |
| *L. yuilli yuilli* | 38.5 | 15.3 | 30.1 | 16.1 | 68.6 | 27.0 | 15.9 | 27.5 | 29.6 | 54.5 | 42.1 | 25.3 | 15.5 | 17.2 | 57.6 | 46.5 | 4.8 | 47.2 | 1.6 | 93.7 |
| *L. youngi* | 38.6 | 16.4 | 28.6 | 16.4 | 67.2 | 26.6 | 15.9 | 27.9 | 29.6 | 54.5 | 42.1 | 25.3 | 15.5 | 17.2 | 57.6 | 47.0 | 8.1 | 42.3 | 2.6 | 89.3 |
| *W. rotundipennis* | 38.0 | 15.6 | 29.6 | 16.8 | 67.6 | 27.9 | 15.0 | 27.6 | 29.5 | 55.5 | 42.5 | 24.5 | 15.9 | 17.2 | 58.4 | 43.6 | 7.3 | 45.3 | 3.8 | 88.9 |
